# A Systematic Evaluation of Blood Serum and Plasma Pre-Analytics for Metabolomics Cohort Studies

**DOI:** 10.3390/ijms17122035

**Published:** 2016-12-05

**Authors:** Elodie Jobard, Olivier Trédan, Déborah Postoly, Fabrice André, Anne-Laure Martin, Bénédicte Elena-Herrmann, Sandrine Boyault

**Affiliations:** 1 Univ Lyon, CNRS, Université Claude Bernard Lyon 1, ENS de Lyon, Institut des Sciences Analytiques UMR 5280, 5 rue de la Doua, F-69100 Villeurbanne, France; elodie.jobard@ens-lyon.fr; 2 Centre Léon Bérard, Département de Recherche Translationnelle et de l’Innovation, 28 rue Laënnec, 69373 Lyon, CEDEX 08, France; 3 Centre Léon Bérard, Département d’oncologie Médicale, 28 rue Laënnec, 69373 Lyon, CEDEX 08, France; olivier.tredan@lyon.unicancer.fr; 4 Centre Léon Bérard, Département de Recherche Translationnelle et de l’Innovation, Génomique des Cancers, 28 rue Laënnec, 69373 Lyon, CEDEX 08, France; deborah.postoly@lyon.unicancer.fr (D.P.); sandrine.boyault@lyon.unicancer.fr (S.B.); 5 Department of Medical Oncology, Gustave Roussy, Université Paris-Saclay, 94805 Villejuif, France; fabrice.andre@gustaveroussy.fr; 6 R&D UNICANCER, 101 rue de Tolbiac, 75654 Paris, CEDEX 13, France; al-martin@unicancer.fr

**Keywords:** metabolomics, pre-analytics, nuclear magnetic resonance, serum, plasma, quality control

## Abstract

The recent thriving development of biobanks and associated high-throughput phenotyping studies requires the elaboration of large-scale approaches for monitoring biological sample quality and compliance with standard protocols. We present a metabolomic investigation of human blood samples that delineates pitfalls and guidelines for the collection, storage and handling procedures for serum and plasma. A series of eight pre-processing technical parameters is systematically investigated along variable ranges commonly encountered across clinical studies. While metabolic fingerprints, as assessed by nuclear magnetic resonance, are not significantly affected by altered centrifugation parameters or delays between sample pre-processing (blood centrifugation) and storage, our metabolomic investigation highlights that both the delay and storage temperature between blood draw and centrifugation are the primary parameters impacting serum and plasma metabolic profiles. Storing the blood drawn at 4 °C is shown to be a reliable routine to confine variability associated with idle time prior to sample pre-processing. Based on their fine sensitivity to pre-analytical parameters and protocol variations, metabolic fingerprints could be exploited as valuable ways to determine compliance with standard procedures and quality assessment of blood samples within large multi-omic clinical and translational cohort studies.

## 1. Introduction

The parallel development of biobanking and high-throughput sequencing, genotyping and phenotyping technologies has enabled a new generation of successful molecular epidemiology studies, such as genome-wide association studies (GWAS) [1], metabolome-wide association studies (MWAS) [2,3] and even metabolomics GWASes [4,5,6]. It currently provides a wide range of new opportunities for the development of biomarkers of medical interest with current applications in toxicology [7], cancer [8,9,10], cardiovascular disease [11,12], prediction of treatment outcomes [13,14,15] or “pharmacometabonomics” [16,17], and more recently metabolic modelling of the patient journey in a clinical environment [18,19]. Untargeted metabolomics is typically achieved using a range of analytical technologies, such as mass spectrometry (MS) and nuclear magnetic resonance (NMR) [20,21,22], resulting in the monitoring of hundreds to thousands of molecular species potentially involved in the molecular fingerprints of diseases.

For NMR, a range of high-level biomarkers discovery tools is available to identify novel compounds involved in disease signature. Biomarkers, in order to be valuable, need to be immune to uncontrolled variation in the technical handling of the samples, vary minimally over the short term for a given individual, and present clear associations with disease risks, progression or response to treatment, or environmental exposures. Mining of large datasets, such as complex NMR metabolic profiles, is inevitably associated with multiple statistical testing, resulting in an increased risk of spurious conclusions if great care is not taken to eliminate bias and minimize uncontrolled experimental variance. While the parallel progress in biobanking and high-throughput sequencing/phenotyping opens a wide range of new research opportunities, they consequently require both standardized protocols and large-scale monitoring of samples quality.

Serum and plasma are dynamically regulated and their compositions progressively change ex vivo as many of the components are not stable and subject to oxidation, aggregation, or degradation. Poorly defined pre-analytical procedures may be a major source of variability and artefacts. In metabolomics, the influence of the pre-analytics practices for blood samples have been widely studied over the last few years [23,24]. For NMR-based metabolomics, it was demonstrated for plasma that ethylenediaminetetraacetic acid (EDTA), heparin or citrate anticoagulants can heavily influence metabolic information recovery [25,26,27,28,29]. Similarly, the UK biobank showed that storage of serum spectra at 4 °C for 24 h before freezing significantly affects the NMR-based metabolic profile [25]. Clot time, clot temperature as well as the number of freeze-thaw cycles have also been shown to influence NMR-based metabolic profiles for blood serum [23,26,30,31,32,33]. Despite the definition of standardized protocols, a strict execution of these protocols within single- or multi-center clinical studies can be challenging when dealing with continuous fluxes of samples, and analytical approaches to monitor the compliance to best practices or to qualify samples available from biobanks are yet to be implemented at the large-scale.

In this work, we investigate a series of technical parameters that may impact high-throughput molecular phenotyping when poorly controlled in clinical research settings. These parameters are precisely controlled and varied and NMR-based metabolomics profiling is carried out for each considered variant condition. Data analysis identifies the major experimental causes of phenotypic variations that allow delineation of precise guidelines for sample collection, storage and subsequent handling of serum and plasma intended for high-throughput molecular phenotyping investigations. We suggest that metabolomic analysis could also provide a relevant set of biomarkers or a global metabolic fingerprint adequate to eventually select serum and plasma samples provided by biobanks or clinical centres, and qualify them for large cohort studies.

## 2. Results

Serum and plasma were analyzed to probe the effect of blood pre-analytics common practices by comparing metabolic profiles obtained after following well-defined reference and variant protocols. First, the delay (dead time) and conservation temperature of the samples between blood draw and centrifugation were examined both individually and concurrently to monitor their impact on the blood metabolome as well as their optimal combination. Two temperature conditions (22 °C and 4 °C) were considered, corresponding to samples left idle on the workbench or preserved in a fridge for a delay of either 1 or 6 h prior to centrifugation. Three variants protocols were thus obtained (Vp1: 1 h at 4 °C, Vp2: 6 h at 4 °C and Vp3: 6 h at 22 °C) in addition to the reference protocol (Ref: 1 h at 22 °C) (Table 1).

**Figure 1:**
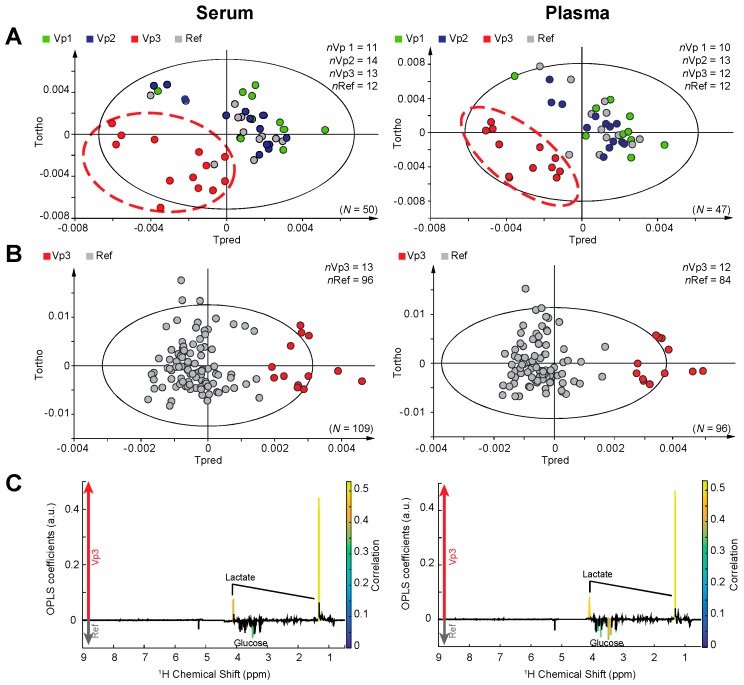
Impact of the delay and storage temperature between blood draw and centrifugation on plasma and serum metabolic profiles. (**A**) Partial least squares discriminant analysis (PLS-DA) model for serum cohort, discriminating variant (Vp) 1, Vp2, Vp3 and a reference (Ref) group (*N* = 50, *n*Vp1 = 11, *n*Vp2 = 14, *n*Vp3 = 13, *n*Ref = 12, 1+1 components, R^2^Y = 0.267, Q^2^ = 0.192) and for plasma cohort (*N* = 47, *n*Vp1 = 10, *n*Vp2 = 13, *n*Vp3 = 12, *n*Ref = 12, 1+1 components, R^2^Y = 0.283, Q^2^ = 0.204). The Ref group corresponds to a random mix of samples collected according to the reference protocol Patients in the Ref group also underwent the variant protocol Vp1, Vp2 or Vp3; (**B**) Orthogonal projections to latent structures (OPLS) model for serum cohort, discriminating Vp3 vs. Ref samples (*N* = 109, *n*Vp3 = 13, *n*Ref = 96, 1+2 components, R^2^Y = 0.7, Q^2^ = 0.652, ANOVA of the cross-validated residuals (CV-ANOVA) *p*-value = 2.7 × 10^−21^); OPLS model score plot for plasma cohort, discriminating samples Vp3 vs. Ref samples (*N* = 96, *n*Vp3 = 12, *n*Ref = 84, 1+2 components, R^2^Y = 0.796, Q^2^ = 0.771, CV-ANOVA *p*-value = 1.9 × 10^−26^); (**C**) OPLS loading plot is represented for Vp3 vs. Ref serum samples; OPLS loading plot is represented for Vp3 vs. Ref plasma samples. Statistically significant individual signals correspond to the color spectral regions. Tpred and Tortho correspond to the predictive component and orthogonal component of the OPLS model, respectively.

Partial least squares discriminant analysis (PLS-DA; 4 classes) was carried out on the NMR metabolic profiles obtained for the same set of patients following these four protocols, and the corresponding score plots show a clear clustering of Vp3 samples in serum and plasma with respect to other groups of samples (Figure 1A). Furthermore, a clear discrimination between Vp3 (13 patients) and all Ref samples (96 patients) of serum (R^2^Y = 0.7, Q^2^ = 0.652, ANOVA of the cross-validated residuals (CV-ANOVA) *p*-value = 2.7 × 10^−21^) and plasma (R^2^Y = 0.796, Q^2^ = 0.771, CV-ANOVA *p*-value = 1.9 × 10^−26^) metabolic profiles is observed from orthogonal partial least squares discriminant analysis (OPLS-DA), as illustrated in Figure 1B. Statistical significance of these OPLS models is assessed by high values of goodness-of-fit parameters R^2^ and Q^2^, CV-ANOVA *p*-values < 0.05 and model resampling under the null hypothesis (Figure S1A). At room temperature, the degradation process occurs at the time scale of our 6 h intervention, both for serum and plasma.

The absence of significant discrimination between Ref samples and Vp1 or Vp2 samples, as illustrated in Figure S1B,C, shows that storage at 4 °C during idle time after blood collection efficiently slows down the degradation process, which becomes undetectable from a metabolic point of view even when blood samples are left idle up to 6 h before centrifugation. Meanwhile, it may be noted that although an increased serum clot-contact time at room temperature affected Proton NMR (^1^H NMR) metabolic profiles, interindividual variations remained clearly predominant with respect to variations due to pre-analytical parameters, as shown by the first component in principal component analysis (PCA) (Figure S2). An equivalent observation was made in the case of plasma (data not shown).

**Figure 2:**
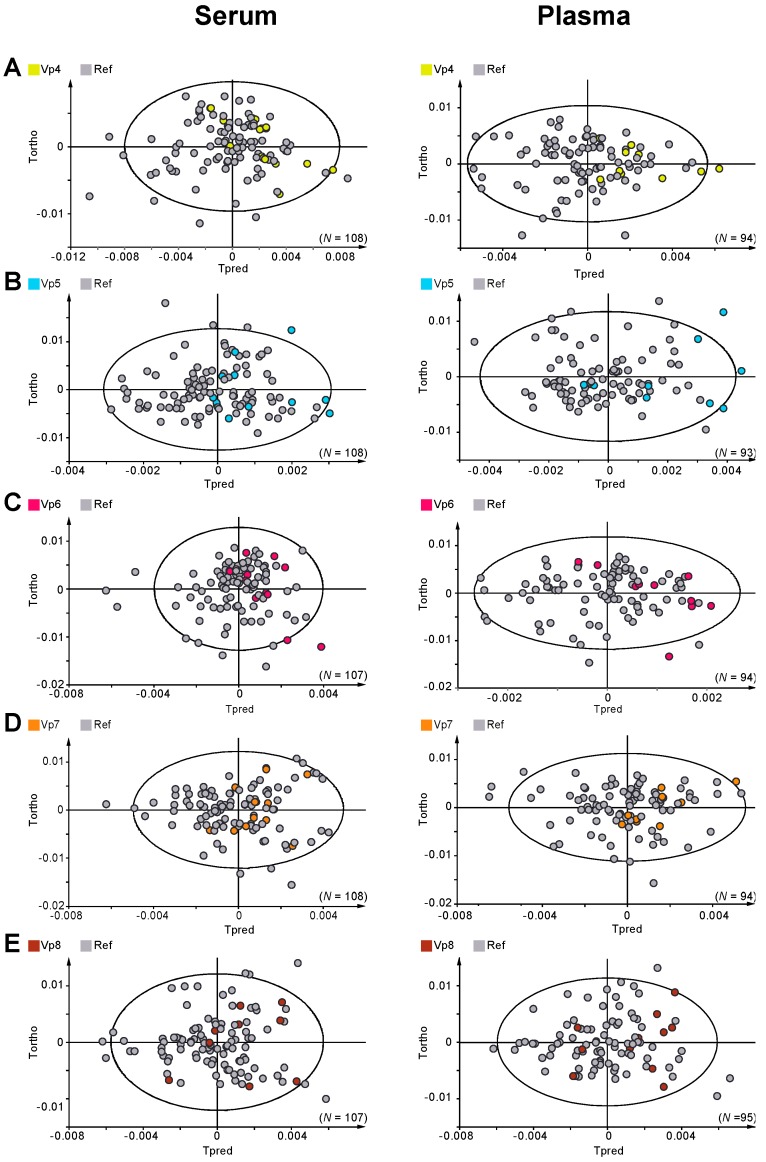
Discrimination between variant protocol samples (Vp4, 5, 6, 7, and 8) and reference protocol samples. (**A**) OPLS models for serum cohort, discriminating Vp4 vs. Ref samples (*N* = 108, *n*Vp4 = 12, *n*Ref = 96, 1+1 components, R^2^Y = 0.069, Q^2^ = −0.146) and for plasma cohort (*N* = 94, *n*Vp4 = 10, *n*Ref = 84, 1+1 components, R^2^Y = 0.148, Q^2^ = −0.019); (**B**) OPLS models for serum cohort, discriminating Vp5 vs. Ref samples (*N* = 108, *n*Vp5 = 12, *n*Ref = 96, 1+1 components, R^2^Y = 0.069, Q^2^ = −0.146) and for plasma cohort (*N* = 93, *n*Vp1 = 9, *n*Ref = 84, 1+1 components, R^2^Y = 0.182, Q^2^ = −0.104); (**C**) OPLS models for serum cohort, discriminating Vp6 vs. Ref samples (*N* = 107, *n*Vp6 = 11, *n*Ref = 96, 1+1 components, R^2^Y = 0.07, Q^2^ = −0.167) and for plasma cohort (*N* = 94, *n*Vp6 = 10, *n*Ref = 84, 1+1 components, R^2^Y = 0.101, Q^2^ = −0.155); (**D**) OPLS models for serum cohort, discriminating Vp7 vs. Ref samples (*N* = 108, *n*Vp7 = 12, *n*Ref = 96, 1+1 components, R^2^Y = 0.031, Q^2^ = −0.111) and for plasma cohort (*N* = 94, *n*Vp7 = 10, *n*Ref = 84, 1+1 components, R^2^Y = 0.053, Q^2^ = −0.06); (**E**) OPLS models for serum cohort, discriminating Vp8 vs. Ref samples (*N* = 107, *n*Vp8 = 11, *n*Ref = 96, 1+1 components, R^2^Y = 0.023, Q^2^ = −0.05) and for plasma cohort (*N* = 95, *n*Vp8 = 11, *n*Ref = 84, 1+1 components, R^2^Y = 0.052, Q^2^ = −0.089).

Centrifugation parameters (rotational speed, temperature and time of centrifugation) were then tested, with no significant difference detected between Ref (at 20 °C) and Vp5 (4 °C) protocols (Figure 2B). Similarly, no effects of the centrifugation time and rotational speed were reflected in the serum/plasma metabolic profiles (Figure 2A,C). In order to study the effects of short-term “storage” of serum and plasma right after centrifugation, the impact associated with waiting times at room temperature of 15 min (Ref) and 1 h (Vp7) on serum and plasma metabolic fingerprints was examined. As shown in Figure 2D, our experimental data present no significant differences between plasma and serum left either 15 min or 1 h spent at room temperature before their freezing at −80 °C. Finally, in order to investigate whether intermediate medium-term storage at −80 °C has an impact on metabolic profiles, we compared plasma and serum stored at −80 °C for 3 ± 1 months (Ref) and 48 ± 24 h (Vp8), respectively. No significant differences were detected between these groups of samples as illustrated in Figure 2E, attesting that blood biofluids are stable at −80 °C for at least 3 months.

**Figure 3:**
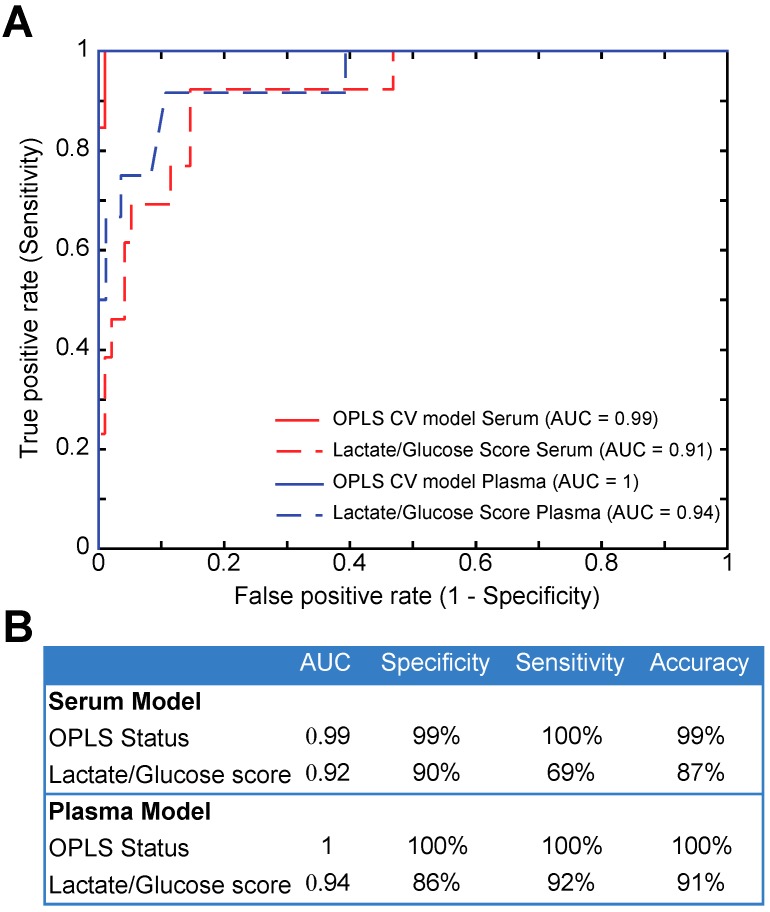
ROC curves analyses. (**A**) Receiver operating characteristics (ROC) analyses including OPLS cross-validated (CV) status and a lactate/glucose ratio for serum and plasma cohort; (**B**) Area under the curve (AUC), specificity, sensitivity and accuracy of the ROC models.

Further to this analysis, individual metabolites significantly associated with the discrimination of Vp3 and Ref samples can be highlighted from univariate analysis of the NMR metabolic profiles, and were identified as lactate and glucose (Figure 1C). When increasing the delay between blood collection and centrifugation for samples kept at room temperature stored, the lactate content increased up to 1.31-fold while glucose simultaneously decreased up to 0.91-fold between Vp3 and Ref serum samples (1.58-fold lactate and 0.83-fold glucose, respectively, for plasma). The performance of the lactate/glucose ratio as a marker of pre-analytical protocol compliance, with respect to the complete multivariate metabolic signature from OPLS-DA, was subsequently assessed by receiver operating characteristic (ROC) analysis. Area under the curve (AUC) for the OPLS and from lactate/glucose classification was 99% and 91%, respectively for serum, and 100% and 94% for plasma samples (Figure 3), consistently demonstrating that global metabolic fingerprints as detected by NMR are more accurate to detect protocol deviations and samples quality than a simple lactate/glucose concentration ratio. Table S1 details the full set of metabolites contributing to the multivariate signature, with notable weights for fatty acids, choline, acetone for both serum and plasma, as well as alanine in the case of serum.

## 3. Discussion

The continuous development of biobanks designed to feed high-throughput molecular sequencing/phenotyping studies requires the development of large-scale strategies for short and long-term monitoring of sample quality. This study, through a systematic intervention of eight independent parameters, identifies in a real-size cohort of human samples both acceptable deviations to standard pre-analytical protocols and sensitive parameters that should be carefully monitored to ensure optimal pre-processing practices. Acceptable deviations from standard protocols shall correspond to those that do not significantly alter the subsequent metabolic profiles. For human blood serum and plasma, we show that only the delay and storage temperature between blood draw and centrifugation have a significant impact on the blood metabolome, while centrifugation parameters (temperature, time and rotational speed), delay between processing and freezing at −80 °C, and short-term storage at −80 °C prior to transfer to liquid nitrogen do not alter the observed metabolic profiles.

To our knowledge, several parameters, such as individual variations of centrifugation times or rotational speeds evaluated both on plasma and serum ^1^H NMR metabolic profiles, are reported here for the first time, while others have previously been investigated on very small sets of samples [25,26,27,28,29,30,31,32]. Several studies have focused on defining standard pre-analytical guidelines for omics investigations. Yet, evaluation of acceptable tolerances to protocol deviations that are inescapable in the context of large multi-center studies and actual clinical infrastructures is still problematic. Transport of samples between collection, processing, and storage facilities is a notable factor not well-accounted for in common standard operating procedures (SOPs), despite being responsible for many protocols nonconformities and little avoidable in authentic studies.

Before centrifugation, alterations of the blood metabolome that are reflected in subsequent serum and plasma profiles are attributable to erythrocyte activity [33]. Our data show that both keeping the processing delay short and maintaining the samples at 4 °C contribute to the efficient preservation of the samples, while the kinetics of the degradation process can still tolerate loosening one of these two parameters. A significant impact on the serum and plasma concentrations of lactate and glucose is only observed when increasing the processing delay to 6 h at room temperature, while serum and plasma profiles of acceptable quality are still obtained for samples kept at 22 °C for 1 h, or at 4 °C for 6 h. Our systematic investigation also shows that a short delay (<1 h) at room temperature between preparation (after centrifugation) and −80 °C freezing has no significant influence on the multivariate metabolomic profiles of serum and plasma, while previous studies have reported a gradual degradation over longer time periods (several hours) for blood samples, notably regarding lipids profiles, in the presence of oxygen at room temperature [26,28,32]. Peculiarly, our results concerning centrifugal parameters do not seem in clear agreement with recent observations from Lesche et al., where changes in the centrifugation protocol (combined variations of centrifugal force and spinning time) significantly influenced plasma metabolomics patterns at room temperature [34]. Under our tested variant protocols, serum and plasma metabolic profiles were shown here invariant to both of these parameters, as well as the centrifugation temperature, when studied individually.

From a different perspective, the sensitivity of the non-targeted metabolomic approach is highlighted here as a relevant sensor for sample quality. In this line, a recent study has proposed the use of a simple quality control marker, the ascorbic acid to lactic acid ratio, as an indicator in EDTA plasma samples of the blood pre-centrifugations conditions, based on gas chromatography–mass spectrometry (GC–MS) analysis [35]. Here, the NMR multivariate metabolic signature discriminating Vp3 and Ref human blood samples displays a better sensitivity and specificity for describing the quality of the sample that a combination of biomarkers (e.g., lactate and glucose) as shown in Figure 3. This metabolic signature and by extension the corresponding OPLS model are robust reporters of the blood human samples lifecycle from the biobank to the analytical end-point. Samples scores on the predictive latent variable (Ref. vs. Vp3) may be used in a broader context to qualify new samples, without relying on any metadata, as acceptable for further metabolomics studies. While metabolic profiles are actually sensitive to pre-analytical protocols and sample mishandling, which can be considered a weakness in the context of large metabolomics investigation, this sharp sensitivity could be exploited in the context of broad “omics” studies where metabolomics signatures could constitute an assessable and cost-effective footprint for the quality control of blood-derived samples.

## 4. Materials and Methods

### 4.1. Design and Sample Collection

For the present study, 96 participants were selected between July 2013 and October 2014, from patients recruited in the large-scale French cohort study cancer toxicities (CANTO – NCT01993498) (12,000 primary breast cancer patients). The local ethics committee (CCP Ile de France VII) approved the research protocol on 14 October 2011. Written informed consent was obtained from each CANTO patient before enrolment. For these participants, 40 mL of blood was drawn at enrollment in the study under fasting conditions. Blood was collected into four 10 mL tubes, either dry or heparin-coated, in order to recover respectively serum and plasma. Each tube was then processed according to a strictly defined and independent protocol. Four different samples (two serum and two plasma) were ultimately obtained per participant. For each individual, one serum and one plasma tubes were processed according to the CANTO standard protocol (Ref) and define the respective serum and plasma control groups. Additional serum and plasma samples were processed according to one of the eight variant protocols (Vp1 to Vp8), where either one (Vp1 and Vp3-8), or two (Vp2) pre-processing parameters were precisely varied at a time from the Reference (Table 1). The standard CANTO protocol is defined as follows: samples are kept at room temperature (22 °C) for 60 min after collection (to ensure complete coagulation in the case of serum), then centrifuged at a speed of 2000× *g* for 10 min at 20 °C. Once centrifuged, samples are immediately (within 15 min) frozen at −80 °C, and stored at −80 °C for 3 months prior to transfer to liquid nitrogen. To define variant protocols, a total of eight pre-processing technical parameters were identified for their potential impact on serum/plasma composition: the delay and preservation temperature between blood draw and centrifugation, the duration, temperature and acceleration of the centrifugation step, the delay between the end of centrifugation and freezing at −80 °C, and finally the duration of conservation at −80 °C prior to storage at −196 °C. These parameters, and their respective amplitudes of induced variation, were selected as those being the most likely to occur in the context of a clinical research project. A total of 192 serum samples and 189 plasma samples were in the end available for our NMR metabolic profiling investigation. Detailed numbers of samples per group (reference and variant protocols) are provided in the Table S2.

### 4.2. Sample Preparation

For NMR analysis, each serum and plasma sample was prepared according to the standard protocol from manufacturer (Bruker GmbH, Rheinstetten, Germany). Samples were thawed at room temperature before use. 200 µL of each was diluted with 400 µL of a buffer solution (0.142 Na_2_HPO_4_ *w*/*v*, NaN_3_ 4% *v*/*v*, D_2_O/H_2_O 10% *v*/*v*) in a microtube. Samples were then centrifuged for 5 min at 4 °C and 12,000× *g*. Finally, 550 µL of supernatant was transferred into 5 mm NMR tubes. Samples were kept for less than 24 h at 4 °C until NMR analysis. To monitor the good reproducibility of NMR data acquisition over time, additional quality control (QC) samples were prepared according to the same protocol. Serum (respectively plasma) QC samples were obtained by aliquoting serum (respectively plasma) from one healthy blood donor provided by Etablissement Français du Sang (EFS), Lyon, France.

### 4.3. ^1^H NMR Spectroscopy

All NMR spectra were recorded on an Avance III spectrometer (Bruker) operating at 600.55 MHz for proton, equipped with a 5 mm cryo-cooled triple resonance probe, and automatic sample changer with a cooling rack at 4 °C. The temperature was then regulated at 27 °C for serum and 37 °C for plasma throughout the NMR experiments. Independent NMR acquisition sessions were carried out for the respective sets of serum and plasma samples. For each session, automatic 3D shimming was performed once at the beginning on a serum/plasma QC sample. In practice, two QC serum/plasma samples were introduced respectively at the beginning and the end of each samples rack corresponding to one day of NMR throughput (~40 samples per day) in order to evaluate both the variability between the first and the last samples of the racks together with the reproducibility over the whole experimental session. Prior to NMR data acquisition, automatic tuning and matching, frequency locking on D_2_O and 1D automatic gradient shimming were performed on each sample. Standard ^1^H 1D NMR pulse sequences, nuclear Overhauser effect spectroscopy (NOESY) and Carr–Purcell–Meiboom–Gill (CPMG) with water presaturation, were applied on each sample to obtain the corresponding metabolic profile. A total of 128 transient free induction decays (FID) were collected for each experiment into 36,010 points over a spectral width of 12 kHz (20 ppm). For both sequences, the total acquisition time was 1.49 s with relaxation delay of 2 s, and the 90° pulse length was automatically calibrated for each sample at around 11.7 µs with a power level of 6 W. The NOESY mixing time was set to 10 ms and the CPMG spin-echo delay to 300 µs (for a total T2 filter of 15.6 ms) allowing an efficient attenuation of the lipid NMR signals. All FIDs were multiplied by an exponential weighting function corresponding to a 0.3 Hz line broadening factor, prior to Fourier transformation. All spectra were referenced to the anomeric proton signal of α-glucose (doublet at δ = 5.23 ppm). ^1^H NMR spectra were phased and baseline was corrected using Topspin 3.1 (Bruker GmbH). Prior to statistical analyses, 19 out of 189 out of plasma samples were excluded due to their poor spectral quality. The final sample set available for further data analysis therefore included 192 serum and 170 plasma samples. After importing all 1D spectra into the AMIX software (Bruker GmbH), spectra were divided into 0.001 ppm-wide buckets to obtain 8500 buckets over the chemical range of 0.5–9 ppm. The variables range corresponding to residual water signal (for serum spectra: 4.4 to 5.11 ppm and for plasma spectra: 4.2 to 5.11 ppm) was excluded. Spectra were normalized to their total intensity and the data matrix was centered. The mean CPMG spectra of serum and plasma samples are presented in Figure S3. In addition, 2D NMR experiments (^1^H-^13^C HSQC, ^1^H-^1^H total correlation spectroscopy (TOCSY) and J-Resolved experiments) were recorded on a subset of samples to achieve structural assignment of the metabolic signals. The procedure for metabolite identification exploits knowledge from academic spectral databases such as Human Metabolome Database (HMDB) [36], as well as proprietary databases (Chenomx NMR Suite v. 7.1, Chenomx Inc., Edmonton, AB, Canada; AMIX Spectral Base v. 1.1.2, Bruker GmbH).

### 4.4. Multivariate and Univariate Analysis

Multivariate analyses of the NMR data were conducted using either PCA, or supervised statistical multivariate methods (PLS or OPLS) to build models for sample classification and extract group-specific metabolic signatures. They were conducted using SIMCA-P 13 (Umetrics, Umea, Sweden). Score and loading plots were used to visualize the data. For the score plot, each point represents a NMR spectrum (i.e., a sample) on the main principal components, and the loading plot visualizes the contribution of the NMR spectral buckets (i.e., metabolic variables) to principal components. PCA was first carried out on the full set of samples, including QCs, respectively for serum and plasma to assess the consistency of the CPMG NMR dataset and reproducibility of measurements over time (Figure S4). Supervised methods were then performed to discriminate the tested pre-processing protocols by using a supplementary data matrix Y, containing information about the protocols, to the X NMR dataset matrix [37]. The goodness-of-fit parameters R^2^ and Q^2^, which relate respectively to the explained and predicted variance, evaluate the OPLS model performance. For each OPLS model, a model validation in MATLAB R2016b (The MathWorks Inc., Natick, NA, USA), using homemade OPLS routines, was performed by resampling the model 1000 times under the null hypothesis through random permutations of the Y matrix. The decrease of goodness-of-fit R^2^ and Q^2^ parameters, when correlation between original model and random models decreases, indicates the good quality of our model. The statistical significance of the calculated model is also assessed for each model by CV-ANOVA (*p*-value < 0.05) [38]. To derive statistically significant associations of individual metabolites, a univariate methodology previously described that couples an automatic binning procedure named statistical recoupling of variables (SRV) to subsequent ANOVA analysis and multiple testing correction of the *p*-values was used, implemented with MATLAB homemade routines [39]. Variable importance in the prediction (VIP) values were exploited to evaluate respective contributions of all metabolites in the metabolic signatures. Variables with a VIP value higher than 1 are considered as important in the model.

### 4.5. Glucose/Lactate Ratio

To determine the glucose/lactate ratio, peak intensities of both metabolites (unambiguously identified and with minimal overlap, glucose: α-doublet to 5.23 ppm, lactate: doublet to 1.32 ppm) were integrated for each sample with AMIX software (Bruker GmbH).

### 4.6. Receiver Operating Characteristics

ROC curves and corresponding AUC were generated for several models including the cross-validated predicted Y values (referred to as OPLS CV) and the lactate/glucose ratio for both serum and plasma samples. The specificity, sensitivity, and accuracy were obtained from the optimal cut-off point, corresponding to the minimal distance to the ideal point (top left corner).

## 5. Conclusions

Our investigation conducted on a large cohort of samples shows that among eight parameters that define standard pre-analytical protocols for human serum and plasma preparation, subsequent metabolic profiles are only significantly affected by the delay and storage temperature between blood collection and sample preparation when systematically reproducing the most common variations encountered across realistic clinical studies. The storage of blood samples at 4 °C for a limited period of time (<1 h) is a conservative and robust strategy to minimize sample evolution associated with anaerobic metabolism of the whole blood. Our findings highlight the sensitivity of metabolomics approaches to detect protocol variations and suggest the metabolic footprint as a global quality probe for evaluation of human blood samples within large clinical and translational omics studies.

## Figures and Tables

**Table 1:** Overview of the study protocol. Fasting blood samples are obtained and handled according to the reference protocol or one of eight variant protocols.

Protocol	Processing					Freezing & Storage	
	Delay of Incubation	Temperature of Incubation	Centrifugation Parameters			Delay between Sample Preparation & Freezing at −80 °C	Time at −80 °C
			Speed	Temperature	Time		
**Reference (Ref)**	**1 h**	**22 °C**	**2000 g**	**20 °C**	**10′**	**15′**	**3 months**
**Variant 1 (Vp1)**	1 h	**4 °C**	2000 g	20 °C	10′	15′	3 months
**Variant 2 (Vp2)**	**6 h**	**4 °C**	2000 g	20 °C	10′	15′	3 months
**Variant 3 (Vp3)**	**6 h**	22 °C	2000 g	20 °C	10′	15′	3 months
**Variant 4 (Vp4)**	1 h	22 °C	2000 g	20 °C	**20′**	15′	3 months
**Variant 5 (Vp5)**	1 h	22 °C	2000 g	**4 °C**	10′	15′	3 months
**Variant 6 (Vp6)**	1 h	22 °C	**3000 g**	20 °C	10′	15′	3 months
**Variant 7 (Vp7)**	1 h	22 °C	2000 g	20 °C	10′	**1 h**	3 months
**Variant 8 (Vp8)**	1 h	22 °C	2000 g	20 °C	10′	15′	**48 h**

## Supplementary Materials

Supplementary materials can be found at www.mdpi.com/1422-0067/17/12/2035/s1.
