# Supplementary material for: A Systematic Evaluation of Blood Serum and Plasma Pre-Analytics for Metabolomics Cohort Studies
Source: Int J Mol Sci. 2016 Dec 5;17(12):2035. doi: 10.3390/ijms17122035 (PMC5187835; doi:10.3390/ijms17122035)
Supplement: Supplementary file 1 [file ijms-17-02035-s001.pdf]

Supplementary Materials: A Systematic Evaluation of Blood Serum and Plasma Pre-Analytics for Metabolomics Cohort Studies

Elodie Jobard, Olivier Trédan, Déborah Postoly, Fabrice André, Anne Laure Martin, Bénédicte Elena-Herrmann and Sandrine Boyault

Table S1. List of metabolic variations discriminating Vp3 vs. Ref samples and corresponding variable importance in the prediction (VIP) values from orthogonal partial least squares (OPLS) analysis. In bold: VIP values > 1 indicate molecules with important weight in the multivariate signature.

Name of Molecule	Direction of Change	Serum Plasma VIP Value	Plasma VIP Value
3-Hydroxybutyrate	−	0.63	0.25
Acetate	−	0.38	0.31
Acetoacetate	−	0.66	0.39
Acetone	+	1.08	1.39
Alanine	+	2.20	0.96
Albumin Lysyl	−	0.17	0.29
Choline	+	2.35	2.45
Citrate	+	0.22	0.22
Creatine	+	0.30	0.22
Creatinine	−	0.33	0.3
FAs (mainly LDL)	+	11.3	11.8
FAs (mainly VLDL)	+	2.72	3.33
FAs	+	6.32	5.30
Formate	−	-	0.05
Glucose	−	3.66	4.81
Glutamate	+	0.41	-
Glutamine	−	0.28	0.48
Glycerol	+	0.52	0.48
PGLYs & TAGs	+	1.03	0.22
Glycerophoshocholine	+	2.35	2.45
Glycine	+	0.64	0.59
Histidine	+	0.13	0.13
Isoleucine	+	0.29	0.28
Lactate	+	30.7	32.2
Leucine	+	0.71	0.23
Lysine	+	0.26	0.25
Methanol	+	0.28	0.21
NAC1	−	0.76	1.11
NAC2	−	-	0.33
Phenylalanine	+	0.07	-
Proline	−	-	0.22
Tyrosine	+	0.09	0.12
Valine	+	0.69	0.68

FAs: Fatty acids; LDL: low-density lipoprotein; NAC: *N*-acetylglycoprotein; PGLYs: Phosphoglycerides; TAGs: Triacylglycerides; VLDL: very-low-density lipoprotein. NAC 1 and NAC2 correspond to NMR signals at 2.03 ppm and 2.07 ppm, respectively. Direction of change: +, corresponds to higher concentration in Vp3 serum or plasma metabolic profiles than Ref samples; −, corresponds to lower concentration in Vp3 serum or plasma metabolic profiles than Ref samples.

Table S2. Numbers of samples per group (reference and variant protocols).

Protocol	Nb of Serum Samples [1]	Nb of Plasma Samples [1]
Ref	96	95 (84)
Vp1	11	11 (10)
Vp2	14	14 (13)
Vp3	13	13 (12)
Vp4	12	12 (11)
Vp5	12	11 (9)
Vp6	11	11 (10)
Vp7	12	11 (10)
Vp8	11	11
Total	192	189 (170)

[1] The outliers are included in table. Between parenthesis is indicated the number of samples without outliers.

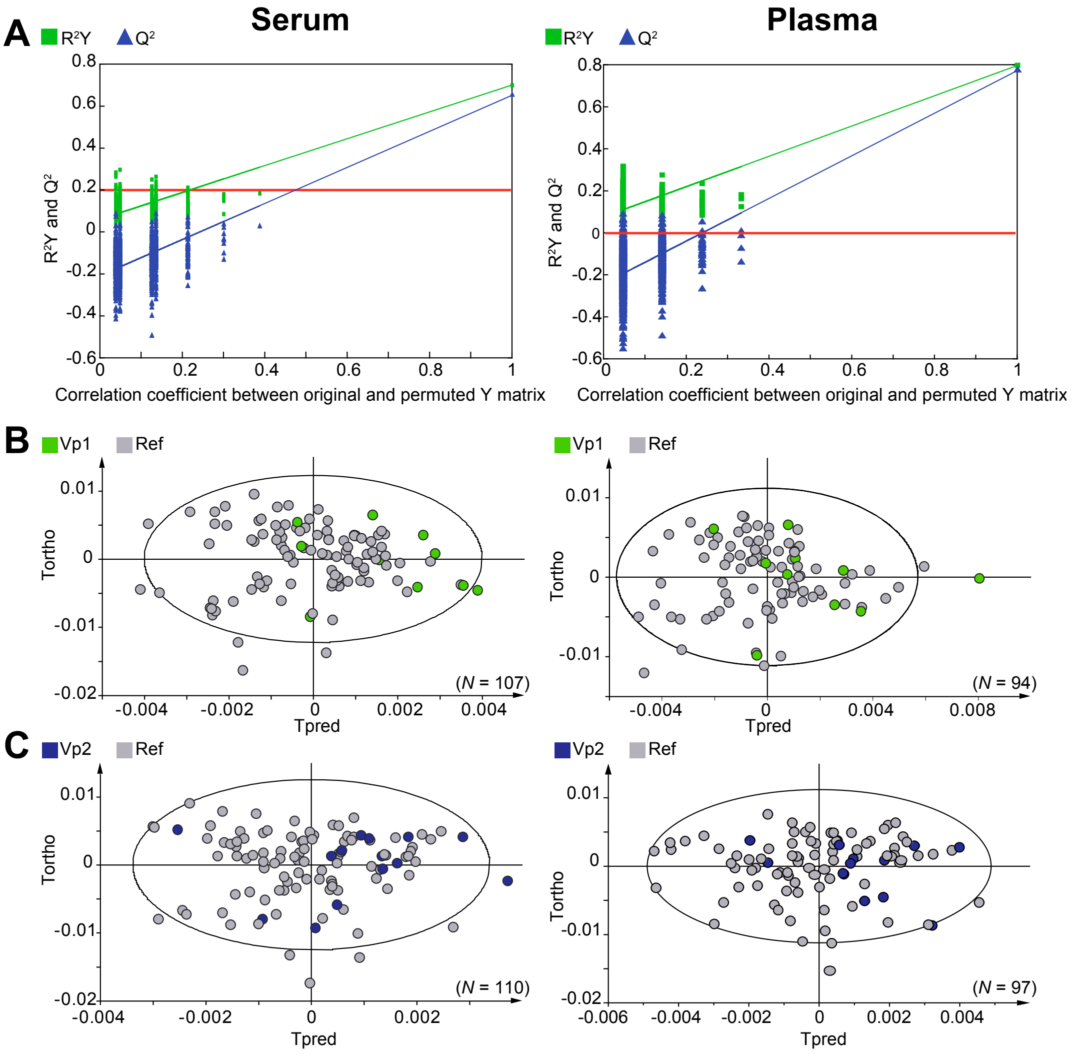

Figure S1. OPLS additional data for Vp1, Vp2 and Vp3. (**A**) OPLS model validations by re-sampling 1000 times the model under the null hypothesis: discriminating Vp3 vs. all standards for the serum cohort and for the plasma cohort; (**B**) OPLS models for serum cohort, discriminating Vp1 vs. Ref samples (N = 107, nVp1 = 11, nRef = 96, 1+1 components, R^2Y = 0.13, Q^2 = −0.141) and for plasma cohort (N = 94, nVp1 = 10, nRef = 84, 1+1 components, R^2Y = 0.06, Q^2 = −0.16); (**C**) OPLS models for serum cohort, discriminating Vp2 vs. Ref samples (N = 110, nVp2 = 14, nRef = 96, 1+1 components, R^2Y = 0.069, Q^2 = −0.143) and for plasma cohort (N = 97, nVp2 = 13, nRef = 84, 1+1 components, R^2Y = 0.058, Q^2 = −0.169).

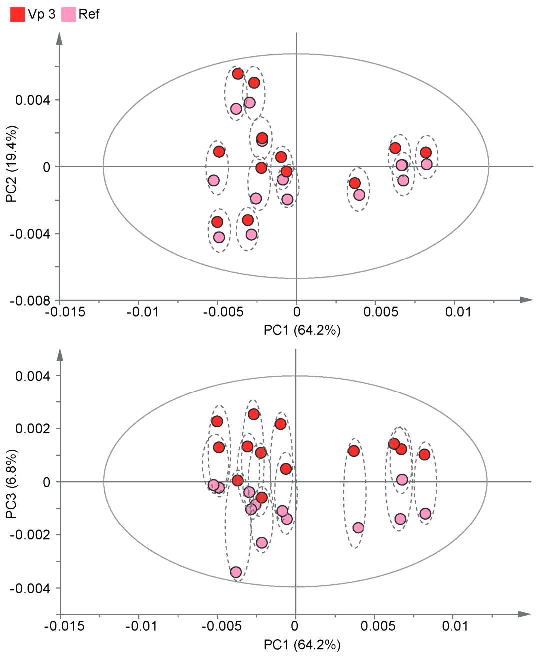

Figure S2. Principal component analysis (PCA) score plots showing the influence of the serum clot-contact time at room temperature. **Top**: PC1 vs. PC2; **Bottom**: PC1 vs. PC3. Serum samples from the same patient are surrounded by dotted lines.

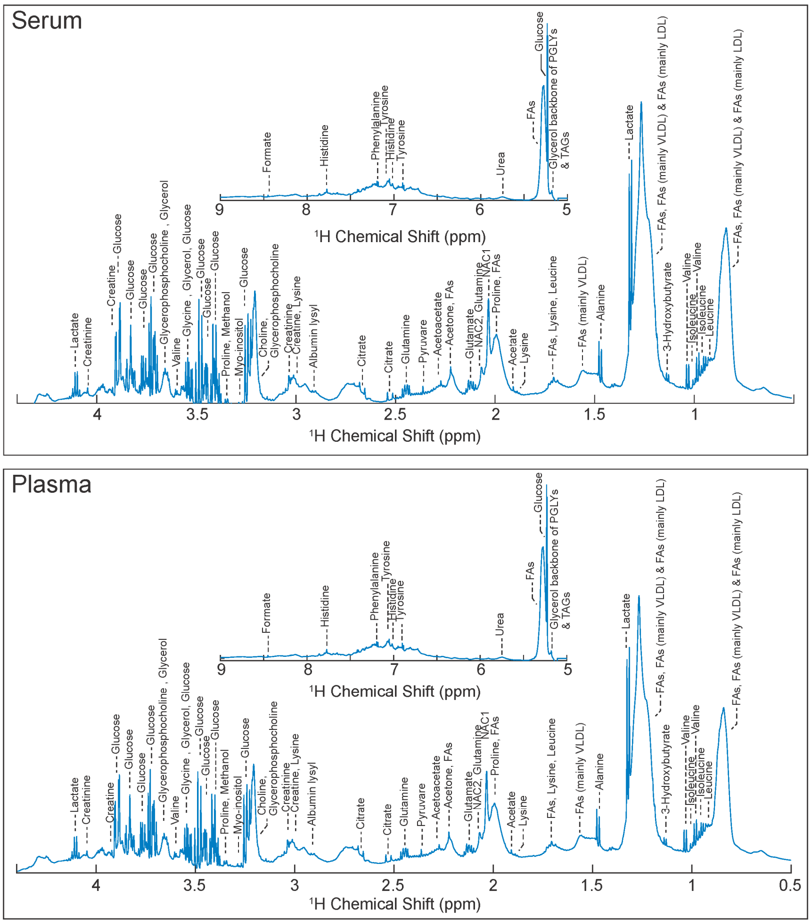

Figure S3. 1H mean Carr–Purcell–Meiboom–Gill (CPMG) spectra (600 MHz) for serum and plasma samples. FAs: Fatty acids; LDL: low-density lipoprotein; NAC: *N*-acetylglycoprotein (NAC1 and NAC2 correspond to NMR signals at 2.03 ppm and 2.07 ppm, respectively.); PGLYs: Phosphoglycerides; TAGs: Triacylglycerides; VLDL: very-low-density lipoprotein.

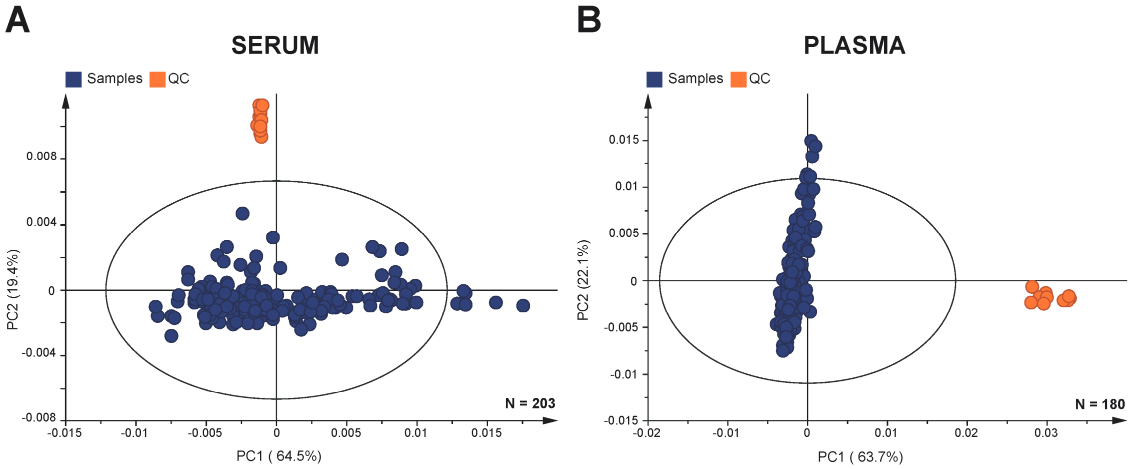

Figure S4. PCA score plots of the ^{1}H NMR CPMG spectra of blood samples acquired at 600 MHz (PC1 vs. PC2). (**A**) N = 203 (nSample = 192, nQC = 11) PC1 = 64.5%, PC2 = 19.4%; (**B**) N = 180 (nSample = 170, nQC = 10) PC1 = 63.7%, PC2 = 22.1%.
